# Long COVID and Myalgic Encephalomyelitis/Chronic Fatigue Syndrome (ME/CFS)—A Systemic Review and Comparison of Clinical Presentation and Symptomatology

**DOI:** 10.3390/medicina57050418

**Published:** 2021-04-26

**Authors:** Timothy L. Wong, Danielle J. Weitzer

**Affiliations:** Department of Psychiatry, Rowan University School of Osteopathic Medicine, Mt. Laurel, NJ 08054, USA; Weitzer@rowan.edu

**Keywords:** long-haul COVID-19, COVID-19, ME/CFS, myalgic encephalomyelitis, chronic fatigue syndrome, systemic review

## Abstract

*Background and Objectives:* Long COVID defines a series of chronic symptoms that patients may experience after resolution of acute COVID-19. Early reports from studies with patients with long COVID suggests a constellation of symptoms with similarities to another chronic medical illness—myalgic encephalomyelitis/chronic fatigue syndrome (ME/CFS). A review study comparing and contrasting ME/CFS with reported symptoms of long COVID may yield mutualistic insight into the characterization and management of both conditions. *Materials and Methods:* A systemic literature search was conducted in MEDLINE and PsycInfo through to 31 January 2021 for studies related to long COVID symptomatology. The literature search was conducted in accordance with PRISMA methodology. *Results:* Twenty-one studies were included in the qualitative analysis. Long COVID symptoms reported by the included studies were compared to a list of ME/CFS symptoms compiled from multiple case definitions. Twenty-five out of 29 known ME/CFS symptoms were reported by at least one selected long COVID study. *Conclusions:* Early studies into long COVID symptomatology suggest many overlaps with clinical presentation of ME/CFS. The need for monitoring and treatment for patients post-COVID is evident. Advancements and standardization of long COVID research methodologies would improve the quality of future research, and may allow further investigations into the similarities and differences between long COVID and ME/CFS.

## 1. Introduction

Coronavirus disease 2019 (COVID-19), a highly contagious respiratory disease caused by the severe acute respiratory syndrome coronavirus 2 (SARS-COV-2), was declared a pandemic by the World Health Organization in March 2020 [1]. As of 7 March 2021, there are over 100 million cumulative cases, with over 2.5 million deaths worldwide [2]. Within the United States alone, there have been almost 30 million cumulative cases, with over half a million deaths as of mid-March [3].

In terms of clinical profile and disease symptomatology, individuals afflicted with COVID-19 vary greatly in terms of clinical presentation [4,5]. While some individuals remain asymptomatic, others experience symptoms generally associated with other viral respiratory diseases, such as fever, cough, dyspnea, headache, and sore throat [6,7,8]. During the acute phase of COVID-19, various other systemic impacts including gastrointestinal, renal, hepatological, rheumatological, and neurological symptoms and complications have been reported [9,10]. While there continues to be significant public concern and research centered around the acute course and presentation of COVID-19, there is increasing public and academic interest in the chronic sequelae of the disease [11,12,13].

There is currently no uniform terminology for this so-called long COVID [14], or, as it has also been termed, long-haul COVID-19 [15,16], post-COVID syndrome [17], chronic COVID syndrome [18], and more recently, post-acute sequelae of SARS-COV-2 infection (PASC) [19]. There is no established case definition or diagnostic criteria, but some have suggested long COVID as being defined by persistent signs and symptoms more than four weeks after initial infection with SARS-COV-2 [20,21]. Research into the prevalence of long COVID is ongoing, but one study has estimated that over 87% of COVID patients continue to experience at least one symptom, two months after COVID symptom onset [22]. The risk for developing long COVID does not appear to be correlated with the severity of acute illness [23]. The etiologies of long COVID are uncertain, with some linking it to autoimmune condition or hyperinflammatory states after resolution of acute COVID [24,25,26].

The characteristics and mysterious nature of long COVID led some to suggest a connection to a debilitating but lesser-known chronic medical condition: myalgic encephalomyelitis/chronic fatigue syndrome (ME/CFS) [27,28,29]. ME/CFS is a long-term complicated illness characterized by at least six months of fatigue and exhaustion. This illness is estimated to account for USD 18–51 billion dollars in economic costs. In total, 2.5 million Americans suffer from chronic fatigue syndrome, with one quarter of those diagnosed being house or bed bound [30]. Within the general population, the prevalence of chronic fatigue ranges between ten and forty percent. Despite this, due to a lack in diagnostic testing without consistent and established treatments, there has been disputes regarding the actual existence of chronic fatigue syndrome. As the diagnosis is mostly based upon patient’s subjective feedback, this has sparked stigma that has led to dismissive behaviors in the medical community. The misconception regarding chronic fatigue syndrome may have been started because of how it was initially characterized. For example, early reports of chronic fatigue were described as a derogatory term known as the Yuppie Flu, which initially characterized the illness among young workers, with the implication of individuals trying to get out of their job responsibilities. However, since this time, the illness has come to be understood to rather affect a broader array of populations, but with a predominance of women being more affected than men [31]. To better understand this illness, improved knowledge of the research and definitions surrounding the illness is needed.

One of the most recent definitions of the illness was formed by the Institute of Medicine in 2015 to avoid further stigma and to promote more knowledge of chronic fatigue syndrome. At that time, the illness was redefined as systemic exertion intolerance disease, with criteria stating that a patient must have significant impairment in the ability to engage in pre-illness levels of educational, occupational, personal, or social activities. This must be due to fatigue that persists for more than 6 months, in addition to post-exertional malaise and unrefreshing sleep, which are other key features of the illness. In addition, the criteria state that a patient must have at least one of the following symptoms: orthostatic intolerance or cognitive decline. As there may be a significant impairment in overall functioning, symptoms should be present with moderate, substantial, or severe intensity with a high frequency of occurrence. These symptoms in chronic fatigue syndrome have been shown to have common onset factors and course. It has been found that during the initial duration of the illness, the most common symptoms were fatigue, pain, cognitive and sleep changes, and flu-related symptoms. As the illness progressed, other medical illnesses tended to worsen the overall course, and few patients had full remission after years of struggle, but rather remained disabled with functional impairments. The most common pattern of onset was following an infectious event, which was followed by gradual progression to consistent sickness. While there have been many theories on the causes of ME/CFS, the three most common precipitating factors have been demonstrated to be infectious illness, stress or major life event, and exposure to an environmental toxin [32]. Several studies have shown that chronic fatigue syndrome patients also react to stressors in an abnormal way, including an abnormal rise in serum cortisol and heart rate in response to the stress of waking up [33].

Over the years, there have been various proposed models for the pathophysiology of ME/CFS. One of the most prominent potential causes of chronic fatigue syndrome includes infection with Epstein–Barr virus (EBV), being highly studied in this setting. A subset of severe chronic fatigue patients exhibit upregulation of EBV-induced gene 2, which serves as a critical gene in immune and central nervous system function. Induction of this gene by EBV could explain the variance of neurological and immune-related symptoms encountered, which has been seen in 38–55% of patients with the illness and has been associated with a variety of autoimmune diseases [34]. Studies have found many other infectious organisms to be associated with chronic fatigue syndrome, including enterovirus, cytomegalovirus, human herpesvirus-6, human parvovirus B19, hepatitis C, *Chlamydophila pneumoniae* and *Coxiella burnetii* [35]. When considering an infectious cause, it will also be important to determine how the human microbiome of persistent pathogens may drive chronic symptoms by interfering with host metabolism, gene expression and immunity. One example of this occurs as bacterial microbes modulate natural killer activity, which has been shown to be reduced in chronic fatigue patients [36]. In terms of immunological explanations, the most consistently reported are increased numbers of activated cytotoxic CD8+T cells and poorly functioning natural killer cells, increased immune activation markers, greater numbers of CD16+/CD3– natural killer cells, and the presence of interferon gamma in serum and cerebrospinal fluid [37]. Other etiologies may include metabolic and endocrine abnormalities, where the body lacks energy and drive because the cells have a problem generating and using energy from oxygen, sugars, lipids, and amino acids. In terms of metabolism, studies have revealed that patients with chronic fatigue syndrome have metabolites including sphingolipids and phospholipids that resemble a hibernation state with significantly lower than normal levels [38].

There exists a large volume of research on the pathogenesis and management of ME/CFS. If long COVID is demonstrated to be a similar chronic medical illness with overlaps in clinical features and symptomatology, it may be conjectured that the existing knowledge on ME/CFS may benefit patients of long COVID. Given the statistics on disease prevalence reported by early studies into long COVID, millions of patients will stand to benefit from insights into the treatment and management of their conditions. Conversely, the increasing public interest in long COVID and an outpouring of research efforts into this condition may yield additional research findings that can benefit patients suffering from ME/CFS. There is an urgent need for studies on the similarities and differences of the symptomatology and pathophysiology of long COVID and ME/CFS. To the authors’ knowledge, there has been no comparative review study into the clinical profiles of long COVID and ME/CFS. Therefore, we conducted a systemic review of the research available thus far into the symptomatology of long COVID, and compared them with known symptoms of ME/CFS based on multiple, widely accepted case definitions.

## 2. Materials and Methods

### 2.1. Search Strategy

We searched MEDLINE and PsycInfo for articles with studies into clinical profiles and symptoms of long COVID, published up to 31 January 2021. As noted in the introduction, due to the lack of uniformed terminologies for long COVID, we used broad, general search terms with the intention of capturing the widest possible array of articles in our literature search.

**Figure 1:**
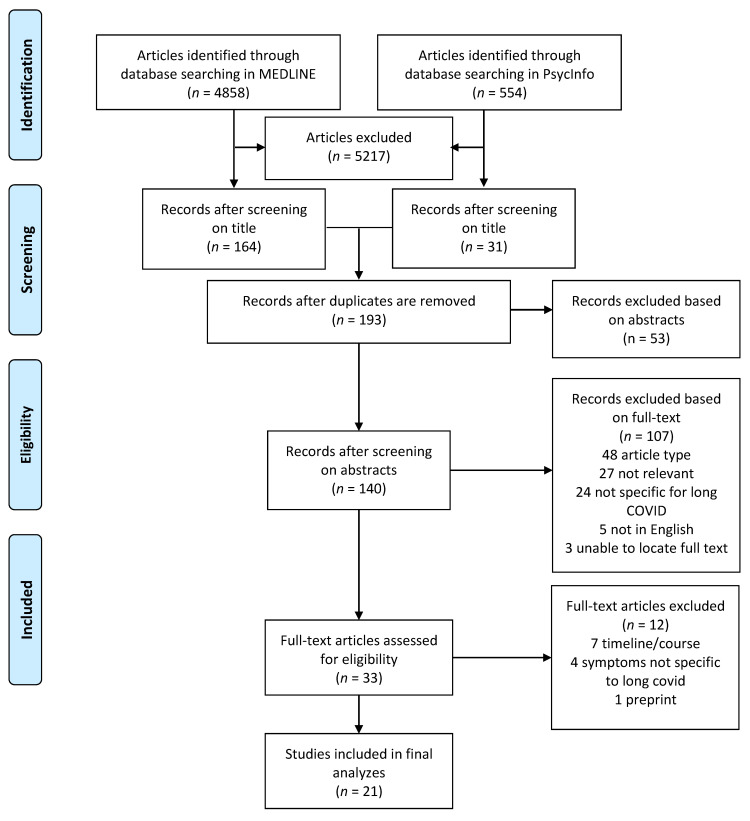
PRISMA flowchart.

For MEDLINE, we used the following search terms: (Long COVID) OR (long haul covid) OR (Chronic COVID) OR (Post-COVID) OR ((“Coronavirus Infections/complications” [Mesh]) AND “COVID-19” [Mesh] AND “Symptom Assessment” [Mesh]). For PsycInfo, we used the following search terms: (Long COVID) OR (long haul covid) OR (Chronic COVID) OR (Post-COVID). The titles and abstracts of the identified articles were reviewed, and the full texts of the selected studies were further examined according to the eligibility criteria. The search and review process are illustrated in Figure 1, following the PRISMA guideline for systemic reviews [39].

### 2.2. Eligibility Criteria

For the purpose of this review article, we required studies with original research data into symptoms of COVID-19, with a clearly defined timeline of at least 4 weeks after the respective study’s reference beginning point, typically time of symptom onset or time of positive COVID test. The reported symptoms must be ongoing at the time of measurement. The symptoms must not be the result of known or identified disease processes that are either self-resolving or resolved with treatment.

### 2.3. Synthesis of Results

We performed qualitative compilation and analysis of data reported by the selected studies. Research into the clinical profile and symptomatology of long COVID is still in its infancy, and there is currently no established methodology or protocol for studying patients with long COVID. Due to the heterogeneity in many aspects of the selected studies, including study populations, data gathering methodologies, and study timelines, a quantitative analysis of the data would be mistaken and inappropriate.

Another consequence of the heterogeneity of long COVID studies and the lack of uniform case definition of long COVID is that the selected studies utilize different terminologies for signs/symptoms that are similar or even identical. As part of the analysis process of study data, we attempted to standardize the terminologies of findings and symptoms by examining the methodologies and measurement methods as described by the corresponding studies, and then further comparing those terminologies to those utilized by ME/CFS case definitions. At times, certain findings and symptoms required further research and interpretation for data analysis. One example would be the 6 min walking test (6MWT) [40], an assessment tool that has been used previously in ME/CFS studies [41,42] and is classified under post-exertional malaise for the purpose of this study.

All long COVID symptoms reported by the selected studies are mapped onto a comparison chart with known ME/CFS criteria. The ME/CFS criteria is adopted from a study by Lim et al. [43] comparing known case definitions of ME/CFS. We selected this compilation of ME/CFS case definitions for our analysis to capture the widest possible array of known ME/CFS symptoms.

## 3. Results

Initially, a total of 5412 articles were identified through database searches through MEDLINE and PsycInfo. After examining the titles and available abstracts of articles, and removing duplicate articles, 140 articles remained. The full texts of the 140 articles were examined and evaluated based on article type and content relevance, and distilled down to 33 articles. The articles were further evaluated based on the eligibility criteria, and 21 articles were selected for the final analysis.

The included long COVID studies are shown in Table 1. The chart further specifies the number of patients included in the studies, patient populations, location, median time at the time of symptom assessment, methodology of assessment, key findings, and other additional findings. The studies are ranked in the chart in descending order according to the number of patients included. As noted in the methods section, the heterogeneity of study methods meant the impracticality of quantitative analysis of the studies; the studies are ranked in this order for the purpose of clarity and qualitative interpretations of the studies. When applicable, the percentage of study patients experiencing long COVID symptoms was provided with the key findings of each study.

The long COVID symptoms described by each study were mapped onto a comparison chart between ME/CFS symptoms, matching long COVID symptoms, and unmatched long COVID symptoms, as seen in Table 2. All except four ME/CFS symptoms (motor disturbance, tinnitus/double vision, lymph node pain/tenderness, sensitivity to chemicals, foods, medications, odors) were reported by at least one selected study on long COVID symptoms. All three major criteria symptoms as specified by most ME/CFS case definitions (fatigue, reduced daily activity, post-exertional malaise) were reported by multiple selected long COVID studies, with fatigue being the most reported symptom (13 out of 21 eligible studies). All sub-categories within the minor criteria of ME/CFS (neurologic/pain, neurocognitive/psychiatric, etc.) were matched with long-COVID studies. Only three selected studies met the ≥6 months duration criteria for ME/CFS. There were a few reported long COVID symptoms that were unique from ME/CFS symptoms, including olfactory dysfunction, gustatory dysfunction, and rash.

## 4. Discussions

To the authors’ knowledge, this is the first review article to examine and compare the symptoms of ME/CFS and long COVID. While there are notable findings when the symptoms reported by the selected long COVID studies were juxtaposed with existing ME/CFS case definitions, it is first worth discussing the quality and design of the selected studies. Though COVID-19 has occupied the public consciousness since the early parts of 2020 [63,64,65], long COVID did not become a subject of public and academic interest until the latter half of 2020, as evidenced by the publication date of the earliest long COVID study included in our paper, July 2020 [22]. The majority of the included studies were published at the end of 2020/beginning of 2021. The research into long COVID is still in its infancy, though there have been ongoing calls for more research and funding into this potentially devastating chronic medical condition [66,67,68].

For the purpose of this systemic review, the implications are such that there is no uniform long COVID case definition, terminologies, and study methods, which leads to a heterogeneity in the study data that precludes quantitative analysis. For one, there is a huge disparity in terms of the timeline of studies, with the time of assessment ranging from a month to 6 months after symptom onset. The studies also vary greatly in assessment methodologies; while some studies utilized patient questionnaires (Goertz et al. [44], Petersen et al. [48], etc.), others utilized in-person evaluations and assessment tools (Townsend et al. [49], Ortelli et al. [56], etc.). Within individual studies, as the authors of one of the studies pointed out, external validity of the studies may be limited due to biases (Goertz et al. [44]). Yet, it is important to keep in mind that the presented studies in this article represent some of the earliest research into symptoms of long COVID, and thus they are hugely valuable in their research into long COVID symptomatology, as well as their insight into future study designs and research protocol into long COVID.

In this systemic review study, the reported symptoms of long COVID from 21 selected studies were compared to a compilation of ME/CFS symptoms from multiple case definitions [43], including Institute of Medicine [69], Fukuda et al. [70], International Consensus Criteria [71], and Canadian Consensus Criteria [72]. The results suggest a high degree of similarities between long COVID and ME/CFS. Out of 29 listed ME/CFS symptoms, all but 4 were reported by at least one long COVID study. It is particularly notable that all three major criteria symptoms, namely fatigue, reduced daily activity, and post-exertional malaise, were reported by multiple studies. Furthermore, fatigue was specifically noted in 12 of the 21 selected studies, which likely suggests fatigue as a predominant symptom of patients suffering from long COVID.

Despite the findings from this comparison, it may be too early to establish a direct causal relationship between long COVID and the development of ME/CFS. Specifically, many of the patients described do not meet the criteria for ME/CFS due to limitations of the studies in regard to duration of symptoms. The diagnosis of ME/CFS requires that the symptoms have been present for at least 6 months. Only three of the selected studies involved the assessment of patients more than 6 months after the onset of their COVID symptoms (Huang et al. [45], Taboada et al. [47], Ludvigsson [57]). The rest of the selected studies range from 37 days to 4 months. It is worthwhile pointing out that within these three studies, 63% of patients from one study reported fatigue (Huang et al. [45]) and 47.5% of patients from another reported decreased functional status (Taboada et al. [47]). In other words, a significant number of patients continue to suffer from long COVID symptoms after 6 months, seemingly at levels comparable to data from studies involving shorter time courses. While the heterogeneity in the study populations and assessment methods preclude meta-analysis of the patient data, it may be suggested that the long COVID symptoms reported by patients in other shorter-duration studies may not resolve completely by 6 months. It has previously been suggested that, even using conservative methodologies, an estimated 10% of patients with COVID-19 may develop chronic illness meeting the definition of ME/CFS [28]. With over 100 million cumulative COVID-19 cases worldwide as of March 2021 [2], the disease burden of this ME/CFS-like chronic illness will likely be devastating.

Aside from similarities in clinical features, long COVID and ME/CFS appear to have certain commonalities in their pathophysiology. As noted in the introduction, the pathogenesis of ME/CFS has been linked to multiple underlying processes including immune system dysregulation, hyperinflammatory state, oxidative stress, and autoimmunity [73]. A particular phenotype of ME/CFS has been termed post-infectious fatigue syndrome, and it has been linked to acute viral infections such as Epstein–Barr virus (EBV) and human parvovirus (HPV)-B19 [74,75]. While the etiology of long COVID is likely multifaceted and the research is still ongoing, it has been similarly linked to inflammatory state and dysregulated immune response [76,77], further underlying the resemblance between long COVID and ME/CFS [78].

Some of the included studies in this review also attempted to characterize the underlying pathophysiology of the long COVID symptoms, and the findings have been mixed. Ortelli et al. [56] noted that their study patients exhibited markedly elevated serum interleukin-6 (IL-6) levels, suggesting the role of hyperinflammation in the pathogenesis of long COVID. However, Townsend et al. [51] did not find any correlation between the patients’ fatigue severity and their serum level of inflammatory markers. Alhiyari et al. [61] noted that the patient in their case report experienced a cough for at least 4 months, and this is likely attributable to the development of pulmonary fibrosis post-COVID. Townsend et al. [49], on the other hand, noted that only a small percentage of their study patients developed pulmonary fibrosis and that this does not appear to be linked with their symptom severity. Further research into the pathogenesis of long COVID and the correlation between acute illness severity and subsequent long COVID symptoms is needed.

With the early research and studies suggesting, at least on certain levels, similarities between the clinical presentation and etiologies of long COVID and ME/CFS, it would be important to consider the implication in the treatment paradigms for both conditions. It would appear that long COVID has so far avoided the earlier obscure fate of ME/CFS, with an outpouring of public and expert acknowledgement for its status as a medical illness and its significant long-term health impact [79,80,81,82]. Many have attested to the importance of providing patient support and monitoring patients’ chronic symptoms post-COVID, as well as the need for further research into long COVID [83,84,85]. Though there is no established treatment protocol for patients with long COVID symptoms, many have acknowledged and suggested the need and benefits of rehabilitation [86,87]. With the consideration that long COVID and ME/CFS may share certain underlying pathological processes, some have suggested that ME/CFS treatment modalities, such as antioxidant therapies, may be beneficial for COVID symptoms [78,88]. In addition, as in the cases with patients with ME/CFS, patients with long COVID may also benefit from developing an energy management plan with a team of interdisciplinary physicians [89]. This may include understanding the patients’ activity threshold and managing daily energy expenditure in order to maintain a healthy active lifestyle while reducing symptom flare-ups. Looking ahead to the future, it may be suggested that the research into long COVID and the ongoing research into ME/CFS may have a symbiotic relationship, with advances made in each medical illness being able to benefit patients suffering from long COVID and ME/CFS.

### Limitations and Future Directions

The results of this review suggest many potential avenues for further exploration and research. While this review provides a qualitative analysis of the similarities and differences between symptoms of long COVID and ME/CFS, a quantitative analysis further delineating the characteristics of both conditions would be warranted. Such an analysis would require further research into the clinical presentation of long COVID, with studies involving standardized methodologies. Investigations into the various contributing factors to long COVID symptoms, including severity of acute disease, history of medical illness, and patient demographics would be beneficial for a future review of ME/CFS and long COVID.

## 5. Conclusions

This review represents the first investigation of its kind into the similarities between symptoms of ME/CFS and long COVID. Based on data from early research into patients suffering from long COVID, this review study suggests many overlaps in the clinical presentation of long COVID and ME/CFS. Further studies into the pathogenesis and symptomatology of long COVID are warranted. With the ever-increasing cumulative cases of COVID-19 worldwide, and the tremendous number of patients who are currently suffering from, or will eventually develop symptoms of long COVID, similar research into long COVID and ME/CFS will be of paramount importance for years to come.

## Figures and Tables

**Table 1:** Studies included in analysis.

	# of Patients	Patient Population	Location	Median Time at Assessment	Methodology	Key Findings	Other Findings
Goertz et al. [44]	2113	Adult; hospitalized + nonhospitalized	Netherlands	79 days after symptom onset	Online questionnaire	Fatigue (87%), dyspnea (71%), chest tightness (44%)	Headache, muscle pain, heart palpitations, cough, sore throat, etc.
Huang et al. [45]	1733	Adult; discharged from hospital	China	186 days after symptom onset	Ambidirectional cohort; questionnaires, etc.	Fatigue/muscle weakness (63%), sleep difficulties (26%)	Anxiety/depression, hair loss, smell disorder, etc.
Mandal et al. [23]	384	Adult; discharged from hospital	U.K.	54 days after discharge	Questionnaire	Fatigue (69%), Breathlessness (53%)	Cough (34%), depression (15%)
Moreno-Perez et al. [46]	277	Adult	Spain	77 days after recovery/discharge	In-person evaluation and questionnaire	Dyspnea (34.4%), cough (21.3%), headache (17.8%)	
Taboada et al. [47]	242	Adult; discharged from hospital	Spain	6 months after discharge	Structured interview	Decreased functional status (47.5%), dyspnea (10.4%)	
Petersen et al. [48]	180	Children + Adult	Faroe Islands	125 days after symptom onset	Patient questionnaire	At least one symptom (53%)	Fatigue, loss of smell and taste, arthralgia, headache, myalgia, dyspnea, etc.
Townsend et al. [49]	153	Adult	Ireland	75 days after diagnosis	Cross-sectional; Chalder Fatigue Scale, etc.	Fatigue (47%)	Decreased performance on six-minute-walk test (6MWT)
Weerahandi et al. [50]	152	Adult; discharged from hospital	U.S.	37 days after discharge	Prospective cohort; PROMIS dyspnea characteristics instrument, etc.	Dyspnea (74.3%)	Worsened mental health
Carfi et al. [22]	143	Adult; discharged from hospital	Italy	60.3 days after symptom onset	Questionnaire	Fatigue (53.1%), dyspnea (43.4%)	Joint pain, chest pain, etc.
Townsend et al. [51]	128	Adult	Ireland	72 days after symptom onset	Chalder fatigue scale	Fatigue (52.3%)	
Halpin et al. [52]	100	Adult; discharged from hospital	U.K.	48 days after discharge	Cross-sectional; telephone questionnaire	Fatigue (64%), breathlessness (50%), PTSD symptoms (31%)	Speech and swallowing dysfunction, continence, vocational difficulties
Wong et al. [53]	78	Adult; discharged from hospital	Canada	3 months after symptom onset	Prospective cohort; questionnaire	Dyspnea (50%), cough (23%)	Anxiety, depression
Le Bon et al. [54]	72	Adult	Belgium	37 days after symptom onset	Prospective cohort; “Sniffin’ Sticks” test battery	Olfactory dysfunction (37%), gustatory dysfunction (7%)	
Woo et al. [55]	18	Adult	Germany	85 days after recovery	TICS-M, fatigue assessment scale, PHQ-9	Cognitive deficits (78%)	Fatigue, mood swings
Ortelli et al. [56]	12	Adult; in neurorehabilitation	Italy	9–13 weeks post COVID	Fatigue rating scale, Beck Depression Inventory, etc.	Neuromuscular fatigue, cognitive fatigue, apathy, executive dysfunction	
Ludvigsson [57]	5	Children	Sweden	6–8 months after COVID onset	Parental report	Fatigue, dyspnea, heart palpitations/chest pain (all 100%)	Headaches, concentration difficulties, muscle weakness, dizziness, etc.
Carroll et al. [58]	1	Adult female	U.S.	50 days after initial infection	Case report	Status epilepticus	
Novak [59]	1	Adult female	U.S.	2.5 months after positive test	Case report	Fatigue, headache	
Koumpa et al. [60]	1	Adult male	U.K.	55 days after symptom onset	Case report	Hearing loss	
Alhiyari et al. [61]	1	Adult	Qatar	4 months after treatment	Case Report	Cough	
Killion et al. [62]	1	Child	Ireland	3 months after hospital admission	Case report	Palmoplantar rash	

**Table 2:** Comparison of compiled ME/CFS symptoms to reported long COVID symptoms.

ME/CFS Criteria	COVID Studies with Matching Symptoms	Non-ME/CFS Criteria Symptoms
**Major criteria**		
Duration ≥6 months	Huang et al., Ludvigsson	
Fatigue	Goertz et al., Huang et al., Mandal et al., Petersen et al., Townsend et al., Weerahandi et al., Carfi et al., Townsend et al., Halpin et al., Woo et al., Ortelli et al., Ludvigsson, Novak	
Reduced daily activity	Huang et al., Taboada et al., Weerahandi et al., Halpin et al., Ludvigsson	
Post-exertional malaise	Huang et al., Townsend et al., Ludvigsson	
**Minor criteria**		
**Neurologic/Pain**		
Myalgia	Goertz et al., Huang et al., Petersen et al., Carfi et al.	
Muscle weakness	Huang et al.	
Motor disturbance		
Generalized hyperalgesia (worsened pain, etc.)	Halpin et al.	
Joint pain	Goertz et al., Huang et al., Petersen et al., Carfi et al.	
Headaches	Goertz et al., Huang et al., Moreno-Perez et al., Petersen et al., Carfi et al., Novak	
Sleep difficulties	Huang et al., Mandal et al.	
	Goertz et al., Huang et al., Petersen et al., Carfi et al., Le Bon et al.	Olfactory dysfunction
	Goertz et al., Huang et al., Petersen et al., Carfi et al., Le Bon et al.	Gustatory dysfunction
	Koumpa et al.	Auditory dysfunction
	Carroll et al.	Seizure
	Halpin et al.	Speech difficulties
**Neurocognitive/Psychiatric**		
Difficulty thinking/processing (brain fog, confusion, etc.)	Moreno-Perez et al., Woo et al., Ortelli et al., Ludvigsson	
Memory difficulties	Moreno-Perez et al., Halpin et al., Woo et al.	
Attention difficulties	Halpin et al., Woo et al., Ortelli et al., Ludvigsson	
Psychiatric (depression, anxiety, PTSD, etc.)	Huang et al., Mandal et al., Weerahandi et al., Halpin et al., Wong et al., Woo et al., Ortelli et al., Ludvigsson	
Hypersensitivity to noise/light	Woo et al.	
Tinnitus, double vision		
**Neuroendocrine**		
Thermostatic instability	Goertz et al.	
Anorexia (loss of appetite, weight loss, etc.)	Goertz et al., Huang et al., Petersen et al., Carfi et al., Halpin et al., Ludvigsson	
**Autonomic Manifestations**		
Orthostatic intolerance (dizziness, etc.)	Goertz et al., Huang et al., Carfi et al.	
Cardiovascular (palpitations, chest pain, etc.)	Goertz et al., Huang et al., Carfi et al.	
Respiratory (dyspnea, etc.)	Goertz et al., Huang et al., Mandal et al., Moreno-Perez et al., Taboada et al., Petersen et al., Weerahandi et al., Carfi et al., Halpin et al., Wong et al., Ludvigsson	
Gastro-intestional (Nausea/vomiting, diarrhea, abdominal pain)	Goertz et al., Huang et al., Petersen et al., Carfi et al., Ludvigsson	
Gastro-urinary (Incontinence, etc.)	Halpin et al.	
**Immune**		
Fever/Chills	Goertz et al., Petersen et al., Ludvigsson	
Flu-like symptoms (cough, etc.)	Goertz et al., Huang et al., Mandal et al., Moreno-Perez et al., Petersen et al., Carfi et al., Wong et al., Alhiyari et al.	
Susceptibility to virus		
Sore throat (swallow problems, etc.)	Goertz et al., Huang et al., Petersen et al., Carfi et al., Halpin et al.	
Lymph node pain/tenderness		
Sensitivity to chemicals, foods, medications, odors		
	Carfi et al.	Sicca Syndrome
**Others**		
	Goertz et al.	Ear pain
	Goertz et al., Moreno-Perez et al., Carfi et al.	Eye problems (red eyes, etc.)
	Goertz et al., Huang et al., Moreno-Perez et al., Petersen et al., Ludvigsson, Killion et al.	Dermatological symptoms (rash, etc.)
	Huang et al.	Hair loss

## Data Availability

Data available upon request.
